# Recommended Immunological Assays to Screen for Ricin-Containing Samples

**DOI:** 10.3390/toxins7124858

**Published:** 2015-11-26

**Authors:** Stéphanie Simon, Sylvia Worbs, Marc-André Avondet, Dobryan M. Tracz, Julie Dano, Lisa Schmidt, Hervé Volland, Brigitte G. Dorner, Cindi R. Corbett

**Affiliations:** 1 CEA Saclay, Institute of Biology and Technologies of Saclay, Laboratory for Immunoanalytical Researches, Gif-sur-Yvette 91191 cedex, France; stephanie.simon@cea.fr (S.S.); julie.dano@cea.fr (J.D.); herve.volland@cea.fr (H.V.); 2 Biological Toxins, Centre for Biological Threats and Special Pathogens, Robert Koch Institute, 13353 Berlin, Germany; worbss@rki.de (S.W.); dornerb@rki.de (B.G.D.); 3 Federal Department of Defence, Civil Protection and Sport—SPIEZ Laboratory, Spiez 3700, Switzerland; marc-andre.avondet@babs.admin.ch; 4 Bacteriology & Enteric Diseases Division, National Microbiology Laboratory, Public Health Agency of Canada, Winnipeg, MB R3E 3R2, Canada; dobryan.tracz@phac-aspc.gc.ca (D.M.T.); lisa.rostek@phac-aspc.gc.ca (L.S.); 5 Department of Medical Microbiology and Infectious Diseases, University of Manitoba, Winnipeg, MB R3E 0J9, Canada

**Keywords:** ricin, immunological detection, enzyme linked immunosorbent assay, lateral flow assay, proficiency test

## Abstract

Ricin, a toxin from the plant *Ricinus communis*, is one of the most toxic biological agents known. Due to its availability, toxicity, ease of production and absence of curative treatments, ricin has been classified by the Centers for Disease Control and Prevention (CDC) as category B biological weapon and it is scheduled as a List 1 compound in the Chemical Weapons Convention. An international proficiency test (PT) was conducted to evaluate detection and quantification capabilities of 17 expert laboratories. In this exercise one goal was to analyse the laboratories’ capacity to detect and differentiate ricin and the less toxic, but highly homologuous protein *R. communis* agglutinin (RCA120). Six analytical strategies are presented in this paper based on immunological assays (four immunoenzymatic assays and two immunochromatographic tests). Using these immunological methods “dangerous” samples containing ricin and/or RCA120 were successfully identified. Based on different antibodies used the detection and quantification of ricin and RCA120 was successful. The ricin PT highlighted the performance of different immunological approaches that are exemplarily recommended for highly sensitive and precise quantification of ricin.

## 1. Introduction

Ricin is a highly toxic protein from the seeds of the castor oil plant (*Ricinus communis*) which has a worldwide distribution, growing naturally across tropical and subtropical regions and is often used as an ornamental plant. Castor beans are used for industrial production of castor oil for hydraulic fluids, paints, and other products [1]. Ricin protein accounts for approximately 1%–5% of an individual castor bean’s weight, which also contains a less toxic but highly homologous protein *R. communis* agglutinin abbreviated RCA120 [2]. Due to the wide availability of castor beans, the straight-forward extraction process to obtain ricin protein, and its high toxicity for potential use as a bioweapon, ricin is classified by the CDC as a Category B select agent [3]. Furthermore, since the toxin has been explored for potential military use by different nations during World War II and later, ricin is the only proteotoxin listed by the Organization for the Prohibition of Chemical Weapons as a controlled chemical under Schedule 1 compounds [4], which prevents the unlawful production, possession, and transfer of ricin toxin. Historically, ricin has been used in previous criminal and bioterrorism attacks (reviewed by Bozza *et al.*, 2015), most notably in the assassination of Bulgarian dissident Georgi Markov in 1978 and mail letter attacks in the United States in 2003 and 2013 [5].

Ricin is an A-B toxin (~60 kDa), consisting of A and B subunits linked by a single disulfide bond. While the B-chain is responsible for binding to different oligosaccharide residues on the cell surface, the ricin A-chain acts as a type 2 ribosome-inactivating protein, depurinating a single adenosine residue in the 28S ribosomal RNA. This adenosine prevents binding of the elongation factor and inhibits protein synthesis, resulting in cell death [6,7]. In contrast to ricin, RCA120 consists of a dimer of two associated ricin-like molecules, each of which contains A- and B-chains (~32 kDa and ~36 kDa) resulting in a ~120 kDa protein [8]; in one publication, a disulphide bond between the two A-chains of RCA120 has been shown by X-ray crystallography [9]. Detection of ricin is complicated by the fact that the A- and B-chains or ricin and RCA120 show a high degree of homology of 93% and 84% on amino acid levels, respectively [10].

Following human exposure via ingestion, injection, or inhalation, the toxic effects of ricin may include nausea, vomiting, dehydration, respiratory failure, and circulatory collapse [1]. Clinical cases of ricin poisoning via ingestion of castor beans occur accidentally or in suicide attempts [11,12,13]. Studies on the toxicity of ricin have been previously reviewed [1,11], and measurements can vary greatly depending on the type of toxicological assessment. The least toxic route of ricin poisoning is via ingestion, with an estimated the lethal dose (LD_50_) of 1 to 20 mg/kg of human body weight [2]. There are cases of self-inflicted ricin toxin injection [14], and the LD_50_ from animal models is 5–10 µg/kg in mice [15] and 3–5 µg/kg in rats [16]. Inhalational ricin toxic effects have an estimated LD_50_ of 3–5 µg/kg [17].

The detection of the ricin protein depends on immunological, mass spectrometry, or functional activity assays [18,19,20,21]. There are many advantages to using immunological-based diagnostic tests for ricin, which have previously demonstrated specificity and sensitivity for ricin toxin. For example, conventional enzyme-linked immunosorbent assay (ELISA)-based methods for detecting ricin toxin have previously been reported with sensitive lower limits of detection (LOD), including: Griffiths *et al.*, (1986), LOD = 20 ng/mL; Leith *et al.*, (1988), LOD = 0.2 ng/mL; Poli *et al.*, (1994), LOD = 0.1 ng/mL; Alderton and Paddle (1997), LOD = 0.01 ng/mL; Shyu *et al.*, (2002a), LOD = 1 ng/mL; and Pauly *et al.*, (2009), LOD = 0.002 ng/mL [22,23,24,25,26,27]. Field-deployable diagnostic tests, such as hand-held lateral flow assay devices (LFA) are another effective immunoassay option for ricin detection, with reported lower limits of detection of 10 ng/mL [28] and 14 ng/mL [29]. Alternative immunoassays for ricin detection include liquid microsphere-based arrays [27], colloidal gold particles [30] and electrochemiluminescence [31]. While the above mentioned immunological methods successfully distinguish ricin from other lectins, the high sequence homology between ricin and RCA120 poses a challenge to immunological methods.

Herein, we describe the immunological detection of ricin and/or RCA120 within four international laboratories during the 2013 EQuATox ricin proficiency panel. For background information on the EQuATox project please see Worbs *et al.* [32]. At the outset of the EQuATox program there was a lack of an internationally accepted ricin toxin standard for analytical detection and identification of ricin within unknown samples. This study represents an important milestone for the acceptance of an international standard material for ricin toxin, as it would enable direct comparison of multiple laboratory approaches for ricin detection and identification. The EQuATox 2013 international proficiency panel consisted of nine blinded samples, containing 1.2 mL liquid samples (S1–S8) which were spiked with ricin toxin and/or RCA120 at various concentrations. Additionally, one solid sample (S9) was distributed that contained a naturally contaminated organic fertilizer containing *R. communis* shred. The task was to analyse the nine samples qualitatively and/or quantitatively with respect to the content of ricin, RCA120 or both related lectins (see Worbs *et al.* [32], this issue of *Toxins*). Both potential analytes were announced at the beginning of the exercise and the choice of methodology to work on this task was free. Additionally, the participants were asked to provide the results of two independent measurements. Considering the restricted volume of sample provided, the laboratories had to carefully plan their analysis strategy.

In this report, we assess the capacity of four out of seventeen participant laboratories to detect, differentiate, and quantify ricin and RCA120 in these proficiency panel samples with immunological assays. The overall proficiency panel test results are discussed in Worbs *et al.* [32], in this issue of *Toxins*.

## 2. Results and Discussion

### 2.1. Overview of Individual Participant Laboratory Assay Validation Studies

**Figure 1:**
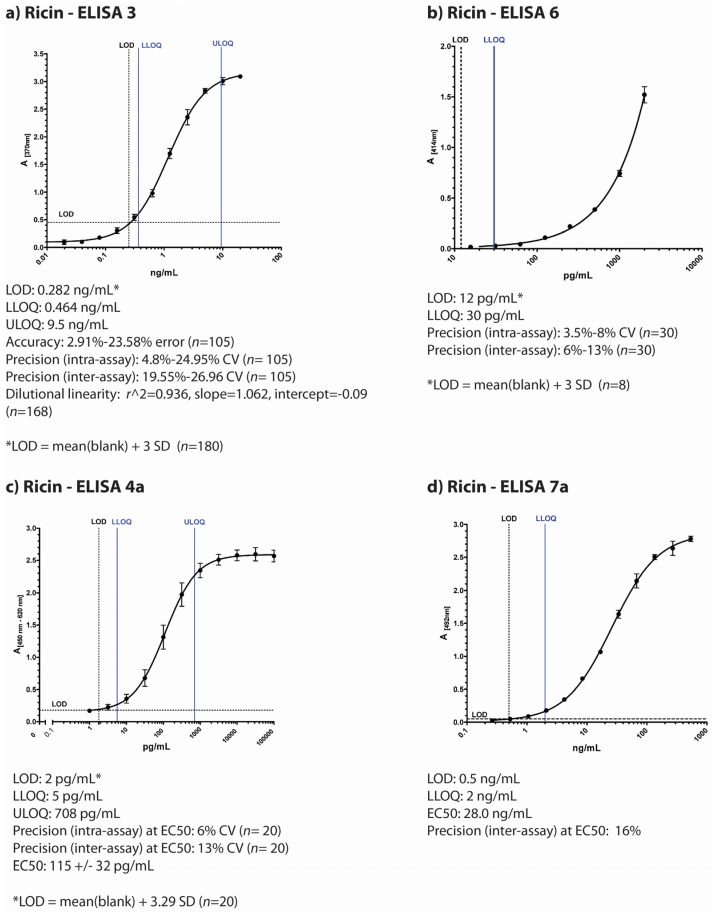

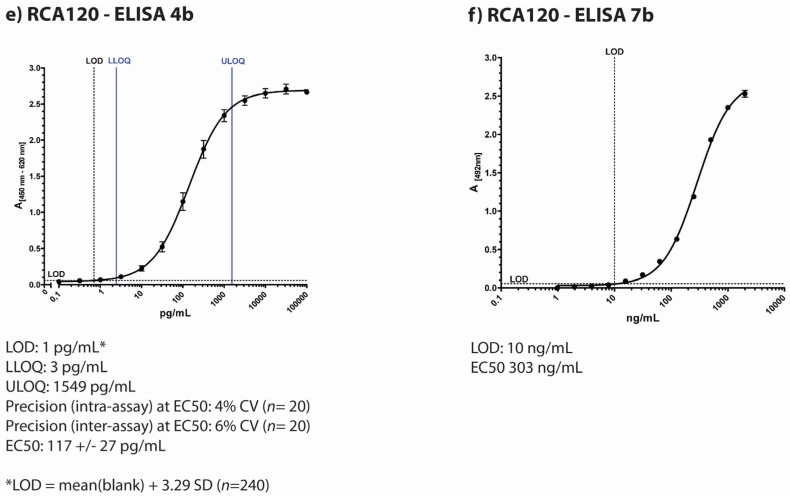
Overview of individual participant laboratory validation studies for different ELISAs detecting ricin and/or RCA120 (**a**) to (**d**) display typical standard curves of four different ELISA used in the ricin PT by four participating institutions based on different antibodies as described in the Experimental Section; (**e**) and (**f**) display standard curves of two ELISA developed for RCA120 detection. The absorption is plotted against concentration of the lectin. Indicated below the pictures are results of the individual validation studies performed by the four institutions prior to the ricin PT. LOD = limit of detection; ULOQ = upper limit of detection; LLOQ = lower limit of detection; SD = standard deviation; *n* = sample size.

Before the PT, each of the immunological approaches applied by the four laboratories underwent an independent, critical validation within the different laboratories addressing similar, but not identical topics. The scope of validation and procedures varied including the use of different ricin (and/or RCA120) standard materials. The results of the in-house validation studies are briefly summarized in Figure 1. Please note the numbering of ELISA- and LFA-formats follows the numbering in Worbs *et al.*, (2015) on ricin PT results this issue of Toxins [32].

#### 2.1.1. ELISA 3

ELISA 3, developed at Public Health Agency of Canada*—*National Microbiology Laboratory (Canada), is an antigen-capture sandwich-type ELISA using monoclonal antibodies (RAC18 and RBC11) that detect both A and B chains of ricin toxin [33] (Figure 1a). This ELISA is validated to ISO 17025 standards as an accredited test in the National Microbiology Laboratory (NML, Canada) quality system. A thorough validation study assessed assay performance characteristics by testing accuracy, inter- and intra- assay precision, dilutional linearity, limits of detection and quantitation, and diagnostic sensitivity and specificity [34]. Accuracy and precision were assessed by testing internal quality control (IQC) standards prepared at known ricin toxin concentrations (using ricin toxin standard prepared in 2009 at RKI, Germany). Data from 23 individual ELISA plates were included in the final data set, which was performed by multiple operators on separate days (see Methods for full description of validation study methodology). The lower limit of detection (LOD) is 0.284 ng/mL of ricin toxin, the empirical lower limit of quantitation is 0.464 ng/mL, and the upper limit of quantitation is 9.5 ng/mL (*n* = 252) [34]. During the validation study, ELISA performance characteristics also demonstrated acceptable levels of intra-assay precision (CV% ≤ 25%, *n* = 105), accuracy (≤25% error, *n* = 105), dilutional linearity (r^2^ = 0.936, slope = 1.062, intercept = −0.09, *n* = 168), as well as diagnostic sensitivity and specificity (100%) for selected plant seed samples, including rosary peas and other seeds.

#### 2.1.2. ELISA 6

ELISA 6 developed at CEA Saclay (France) is a sandwich-type ELISA using monoclonal antibodies (RB37 and RA36) against ricin toxin [35] (Figure 1b). The lower limit of detection of this immunoassay is 12 pg/mL and the cross reactivity with RCA120 is 0.1% (calculated as the percentage of ricin concentration/RCA120 concentration giving the same absorbance in the linear range of the standard curve, 0.5 AU). No cross reaction were observed with other lectins (24 lectins have been tested). The intra-assay coefficients of variation were determined by assaying five times on the same day five different ricin concentrations (30, 50, 100, 300 and 1500 pg/mL). Inter-assay coefficients were determined by repeating this experiment on six different days. Intra-assay coefficients of variation (CV%) were lower than 10% and the inter-assay coefficients variation were below 15% for all the concentrations. As both CVs were below 20% for 30 pg/mL we considered this concentration as the limit of quantification. The reference material used for these experiments was an in-house purified ricin calibrated using its absorbance.

#### 2.1.3. LFA 4

Additionally, CEA Saclay developed an LFA test which is available commercially by NBC-sys (Saint Chamond, France). The test has a limit of detection of 1 ng/mL (using an in-house reference material), with a cross-reactivity for RCA120 of 0.1% (calculated as the percentage of LOD for ricin/LOD for RCA120) and an assay running time of 15 min.

#### 2.1.4. ELISA 4

The Robert Koch Institute, Germany, has developed two different sandwich-ELISA, one preferentially detecting ricin with only little cross-reactivity to RCA120 (Figure 1c) and the other preferentially detecting RCA120 with low cross-reactivity to ricin (Figure 1e; for graphical representation of cross-reactivities please refer to Worbs *et al.* [32]. The ricin-specific ELISA is based on a combination of two monoclonal antibodies and detects both chains of ricin (anti-ricin B chain: mAb R109 and anti-ricin A chain: mAb R18; [27]), while the RCA120-specific ELISA combines a monoclonal with a polyclonal chicken antibody (mAb ARK4 and chicken IgY RC22) [36,37]. In a validation study for the ricin-specific ELISA, the half maximal effective concentration (EC_50_) of the ELISA as point of highest precision with respect to quantification was determined at 115 ± 32 pg/mL with a limit of detection at 2 pg/mL. The working range of the ricin-specific ELISA as the range in which obtained results have a precision of <20% and a trueness of 80%–120% was experimentally determined and a lower and upper limit of quantification was derived at 5 pg/mL and 708 pg/mL, respectively. Intra-assay and inter-assay coefficient of variations were determined at 6% and 13% at the EC_50_ value, respectively, with *n* = 10 as number of intra- or inter-assay repetitions performed in duplicate. No cross-reactivity except for RCA120 to a range of other lectins and toxins was detected. In order to detect ricin in complex matrices, a range of different matrices was spiked with ricin, and high recovery rates were obtained (e.g., 83% for milk and 107% for a meat extract, data not shown). As reference material, an in-house preparation of ricin produced in 2009 was used.

The RCA120-specific ELISA gave similar results in a validation study: The EC_50_ value was determined at 117 ± 27 pg/mL, the LOD at 1 pg/mL. The working range of the RCA120-specific ELISA lay between the lower limit of quantification of 3 pg/mL and the upper limit of quantification of 1549 pg/mL. Intra-assay and inter-assay coefficient of variations were determined at 4% and 6% at the EC_50_ value, respectively (*n* = 20).

#### 2.1.5. ELISA 7

The Spiez Laboratory, Switzerland, uses two different sandwich-ELISAs, one specific for ricin with little cross-reactivity for RCA120 (Figure 1d) and the other preferentially detecting RCA120 with low cross-reactivity to ricin (Figure 1f). The ricin-specific ELISA is based on two monoclonal antibodies RCH1 and 1RK1, both are directed against the A-chain. The procedure has been accredited according to ISO 17025. The validation study included inter- and intra-assay accuracy and precision, linearity, limits of detection and quantification (DIN 32645), batch test of reagents and antibodies, stability tests of antibodies and recovery tests in different matrices. The limit of detection for ricin was determined at 0.5 ng/mL. The lower limit of quantification was about 2 ng/mL with a relative uncertainty of 5%. Within the quantitative working range the coefficient of variation is lower than 15% and the recovery lay between 70 and 130%. The cross-reactivity against the related RCA120 is approximately 1%. During the PT, an experimental ELISA for RCA120 was used that has not yet been validated thoroughly. The ELISA is based on a combination of two mAbs (ARK4 and 1RK1) and provides an EC_50_ value of about 303 ng/mL. At a concentration of 10 ng/mL RCA120 (standard in assay buffer) the coefficient of variation lay under 15% and the recovery rate was between 70% and 130%. By comparing the EC_50_ values of RCA120 and ricin the estimated cross reactivity to ricin was about 20%. An in-house ricin reference material prepared by U. Pfüller (University Witten-Herdecke, Germany) was used.

#### 2.1.6. LFA 2

Additionally, Spiez Laboratory used miPROTECT Ricin test cartridges, an LFA commercially available by miprolab (Göttingen, Germany). The test cartridges include a ricin specific colloidal gold-labled mAb and a glycoprotein for capturing. The limit of detection was around 20 ng/mL validated using the in-house reference material. A previous validation study showed that the LFA detected both ricin and RCA120. 

### 2.2. Overview of the Diagnostic Approaches Utilized by the Participating Laboratories

**Figure 2:**
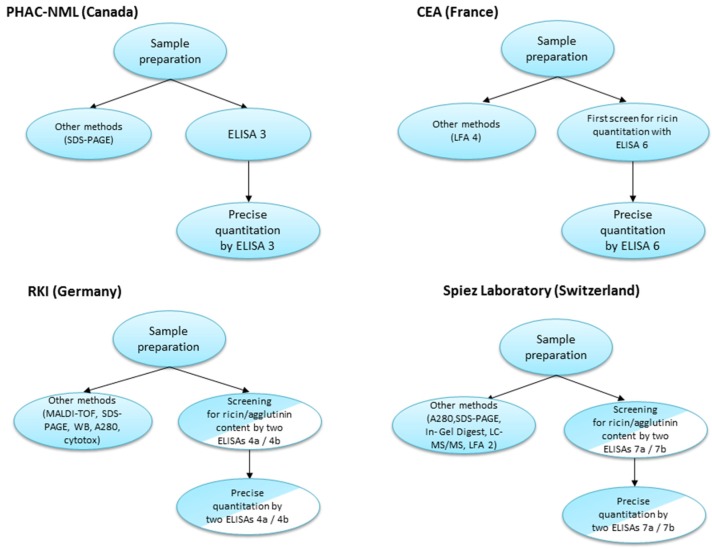
Comparison of diagnostic approaches utilized by the laboratories during the 2013 EQuATox ricin proficiency test.

The four participating laboratories contributing to this manuscript utilized various approaches for sample analyses during this proficiency study. A schematic overview of each process is represented in Figure 2.

As observed above, each of the laboratories applied a different diagnostic approach for sample analyses (Figure 2). Of the nine samples, liquid samples S1–S8 contained a volume of 1.2 mL volume, whereas for sample S9 10 g of solid material were provided. The limiting factor of sample volume restricted the number and the quality of analyses that could be performed (considering that one of the requirements of the proficiency test was to provide two independent measurements for each sample). 

Canada’s PHAC-NML laboratory performed an initial screen of proficiency panel samples (5 µL) by sodium dodecyl sulfate polyacrylamide gel electrophoresis (SDS-PAGE) to visualize and estimate sample protein content. Based on these estimates, PT panel samples were diluted in assay buffer (for example, S7 was recognized as sample containing low protein content and left neat/undiluted whereas sample S6 was diluted 1/1000 based on the protein content visualized by SDS-PAGE). Samples were serially-diluted 12-fold in assay buffer and 100 µL of each dilution was applied to the assay (ELISA 3), which was repeated for a duplicate measurement on a separate day. Results were compared to a ricin standard curve (with an initial starting concentration of 20 ng/mL), and concentrations were interpolated in the linear range (see Methods). Where necessary, samples were further diluted for subsequent ELISA runs to produce absorbance signals in the linear range of the 4-parameter logistic (4-PL) curve fit, allowing for interpolation of sample concentration. 

For CEA laboratory (ELISA 6), the first screening was realized using sandwich ELISA. Each sample was measured in a log dilution from undiluted to 1:10^3^ dilution in a double determination (use of 450 µL of each sample). In-house purified ricin has been used as standards to determine concentrations of the toxins in the samples. Based on the results obtained in the first screening samples S2, S6, S7 and S9 were retested by sandwich-ELISA from 1:10^2^ to 1:10^5^ dilutions in order to be in the linear range of the standard curve (use of 10 µL of each sample). All the samples have been tested using specific LFA undiluted or diluted 10 fold for samples S4 and S9 (use of 200 µL of each sample and 250 µL for samples S4 and S9). Overall the maximum volume used to perform all the tests (Sandwich ELISA + LFA) for one sample was 710 µL.

RKI used a combined approach applying different principles of detection: In a first screening step, 120 µL of sample were used to perform a ricin (ELISA 4a) and an RCA120-specific ELISA (ELISA 4b). For the two ELISAs, each sample was measured in a log-dilution series from undiluted (50 µL) to 1:10^11^ dilution in a single determination. In parallel, 140 µL of sample were used for matrix-assisted laser desorption/ionization time-of-flight (MALDI-TOF) analysis [19], 11 µL for gel electrophoresis under reducing and non-reducing conditions, 4 µL for photometric measurement of absorption at 280 nm (A280) and 5 µL for analysis by Western blotting (WB). At a later time point, a real-time cytotoxicity assay (100 µL, [18]) and a real-time PCR assay (100 µL) were included. The orienting analysis from the combination of all methods*—*especially the two ELISA and the MALDI-TOF experiments*—*allowed a qualitative assignment of sample contents. Based on these results, it was decided which sample was re-analysed by the ricin- OR RCA120-specific ELISA in duplicate for precise quantitation where 440 µL sample volume were used.

Switzerland’s Spiez laboratory first determined the total protein content (A280, 16 µL per sample) and the samples were grouped into low protein content (S1, S3, S5, S7) and high protein content (S2, S6, S8). Subsequently, more detailed protein information was obtained by SDS-PAGE under reducing and non-reducing conditions (20–30 µL samples). Based on the screening analysis individual sample dilutions were carried out. In minimum a 10-fold dilution was done to save sample material. Then log-dilution series up to 10^4^ to 10^6^ were performed. For final quantification the ricin-ELISA 7a was carried out in duplicate (two independent dilutions on different plates). Based on screening analysis selected samples were measured by diluting to an approximate concentration of 10 ng/mL (*n* = 4 per samples and plate). Sample volumes of 40 µL were used. The low concentration samples (S1, S5, S7) were analyzed undiluted in duplicate (400 µL). For precise quantification of RCA120, two RCA120 ELISAs were performed (ELISA 7b). Based on screening results 40 µL of high concentration RCA120/ricin samples (S2, S6), 160 µL of medium concentration RCA120 sample (S5) and 400 µL of low concentration RCA120 samples (S3, S8) were used. Confirmatory measurements have been made using the ricin LFA (LFA 2) with the samples S2, S3, S4, S6, S8 and S9.

### 2.3. Overall Assessment of Immunological Results

Samples in the 2013 EQuATox proficiency test panel included various matrices and buffers spiked with varying concentrations of ricin and/or RCA120 (Table 1, for an overview on the proficiency test and overall results obtained by different technical approaches please see Worbs *et al.* [32].

**Table 1:** Ricin proficiency test: sample identity and statistics [32].

Sample	Matrix	Measurand	c(Theoretical) *	c(Nominal) **	σ(Rob)	*x_a_* ***	σ_p_	Unit
S1	0.1% BSA/PBS	-	-	-	-	-	-	-
S2	0.1% BSA/PBS	RCA120	500,000	572,851	62,686	563,994	143,876	ng/mL
S3	0.1% BSA/PBS	Ricin	500	504	110	522	133	ng/mL
S4	skimmed milk	Ricin	500	473	96.3	436	111	ng/mL
S5	0.1% BSA/PBS	RCA120	500	445	65.2	481	123	ng/mL
S6	0.1% BSA/PBS	Ricin	500,000	589,508	78,055	588,949	150,242	ng/mL
S7	0.1% BSA/PBS	Ricin	0.5	0.414	0.112	0.441	0.112	ng/mL
S8	meat extract	Ricin	500	484	111	508	130	ng/mL
S9	Organic fertilizer	RCA120	-	42	5.818	42	52.6	µg/g
		Ricin	-	306	71.6	206	10.7	µg/g

* The “theoretical concentration” was the known concentration of ricin or RCA120 that was spiked into the different matrices. Sample S9 was a naturally contaminated material, the true “theoretical values” were not known; ** Robust estimates of mean nominal concentrations as determined experimentally by the organizing laboratory by ELISA for ricin or RCA120, respectively; *** Consensus mean concentration based on all participants’ reported results used as “assigned concentration” *x_a_* are highlighted in green; σ(rob): robust estimate of the standard deviation of the nominal concentrations; σ_p_: standard deviation for proficiency assessment.

#### 2.3.1. Qualitative Results

**Figure 3:**
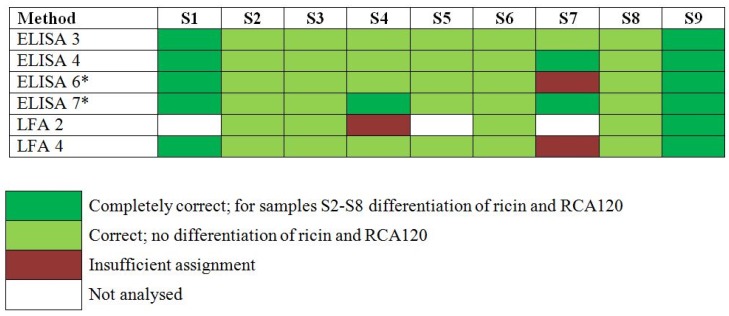
Results of the proficiency test panel, samples S1–S9, by individual participant laboratory method. Sample S1 was the negative control sample, S2 and S5 contained RCA120, S3, S4, S6–S8 contained ricin and S9 was the organic fertilizer containing *Ricinus communis* shred including ricin and RCA120. Qualitative results reported by the participants were color-coded as indicated. * Results have been taken from the laboratory’s quantitative reporting since they accidentally have not been reported qualitatively.

In the PT, participants were asked to report their experimental results as “ricin”, “RCA120”, “ricin and/or RCA120”, “negative result (*i.e.*, nothing detected)” or “not analyzed” in an Excel workbook. The results reported were assessed according to the degree of trueness of the participant’s assignments and color codes were used to indicate the assessment (Figure 3). Samples S2 to S8 were assessed as “correct/light green” if results were reported as “ricin and/or RCA120” without differentiation between ricin and RCA120 and “completely correct/dark green” if differentiation into ricin and RCA120 was successfully performed [32].

Qualitative results (Figure 3) showed that ELISA 3 was correct in identifying ricin and/or RCA120 in all 9 samples of the 2013 EQuATox panel, including S6 with high ricin toxin levels (588949 ng/mL) and S7 with low ricin levels (0.441 ng/mL). Sample S1, a negative control, was correctly found to contain no ricin.

Qualitative results showed that ELISA 6 was correct in identifying 5 of the 6 samples containing ricin (S3, S4, S6, S7, S8, S9). For the sample S7 a misinterpretation of the assays led to an insufficient assignment. Sample S1, a negative control, was correctly found to contain no ricin. The on-site detection assay LFA 4 correctly detected ricin and/or RCA120 in 8 of 9 proficiency panel samples, including sample S6 which had a high concentration of ricin. Only one sample (S7), containing a low concentration of ricin (0.441 ng/mL) yielded an insufficient assignment. In spite of low cross-reaction with RCA120 (0.1%) the LFA was not able to differentiate ricin and large levels of RCA120.

As to approach ELISA 4, results were obtained by using two different ELISA in a first screening step, one preferentially detecting ricin (ELISA 4a), the other RCA120 (ELISA 4b). The qualitative assignment of analytes was correct for all 9 proficiency panel samples: Sample S1 gave negative results in both assays; sample S7 gave signals only in the ricin-specific ELISA (dark green, Figure 3). All other samples resulted in signals of different intensities in both ELISAs, depending on the dilution tested (undiluted to 10^11^). This can be explained by the low, but existing cross-reactivity of both ELISAs. However, based on the strong bias of both ELISAs for either ricin or RCA120, all samples could preliminarily be attributed to contain the one or other analyte except sample S9 which was found to contain significant amounts of both analytes. Still, at this stage a clear differentiation into ricin and RCA120 was not possible by ELISA so that results were reported as “ricin and/or RCA120” for samples S2 to S9 (except S7; light green, Figure 3). For unambiguous identification, MALDI-TOF MS analysis was instrumental to decide which of the two analytes was present in each of the 9 samples. With the MALDI-TOF MS data it was feasible to attribute samples to ricin (S3, S4, S6, S7, S8, and S9) and/or to RCA120 (S2, S5, and S9). Based on these results the corresponding ELISA was used in a second step for precise quantification.

As to approach ELISA 7, similarly two different ELISAs were used to screen for ricin- and/or RCA120-containing samples. Eight samples were tested positive (S2, S3, S4, S5, S6, S7, S8, S9) by the ricin ELISA (ELISA 7a). Sample S1 was identified as the negative control. Six samples (S2, S3, S5, S6, S8, S9) showed positive results by the RCA120 ELISA (ELISA 7b) and samples S7 and S4 were negative. Due to the known cross-reactivity of the RCA120 ELISA five samples (S2, S3, S5, S6, S8) were assigned to contain ricin and/or RCA120. Mass spectrometry (SDS-PAGE, in gel digest and LC-MS/MS) was used in parallel to identify the main compounds in all samples. Ricin and/or RCA120 were identified in samples S2, S6 and S9. Bovine serum albumin was found in samples S1, S2, S3, S5, S6 and S7. In sample S4 different milk proteins were identified. No suitable identification was obtained for sample S8. LFA measurements (LFA 2) were performed in case the amount of ricin was higher than the LOD of the assay (samples S1, S5 and S7 were not tested). All measured samples (except S4) gave positive signals for ricin and/or RCA120. The false negative result for sample S4 was presumably caused by over diluting and maybe a reduced sensitivity due to matrix interference.

Taken together, from the different immunological methods applied in the PT, ELISA 3, 4 and 7 proved to be sensitive to detect all ricin- or RCA120-containing samples offered in the PT. Additionally, LFA 4 was instrumental to detect eight out of nine PT samples correctly.

#### 2.3.2. Quantitative Results

Independent of the qualitative reporting, the participating laboratories were asked to perform quantification of ricin in the nine samples and to report the results of two independent measurements in a dedicated Excel reporting file. Most laboratories used ELISA-based approaches for quantification, some reported results (Table 2) as measurand “ricin” (as in ELISA 4 and 7), others as measurands “ricin and/or RCA120” (as in ELISA 3 and 6). Performance of the selected ELISA with respect to quantification is described below. In order to assess and visualize quantitative results, the PT organizer calculated *z*-scores according to the Equation (1).

(1) $$z=\frac{x-x_{a}}{\sigma_{p}}$$

with *x* denoting the results reported by the participants, *x_a_* the assigned concentration value and σ*_p_* the standard deviation for proficiency assessment, respectively (Table 1 and [32]). *z*-scores quantify the difference between an individual single or mean result and the assigned value in units of the standard deviation for proficiency assessment. A *z*-score of zero indicates an unbiased result with respect to the assigned value, a *z*-score of 1 is one standard deviation for proficiency assessment above the assigned value, a *z*-score of –1 is one standard deviation for proficiency assessment below the assigned value and so on.

**Figure 4:**
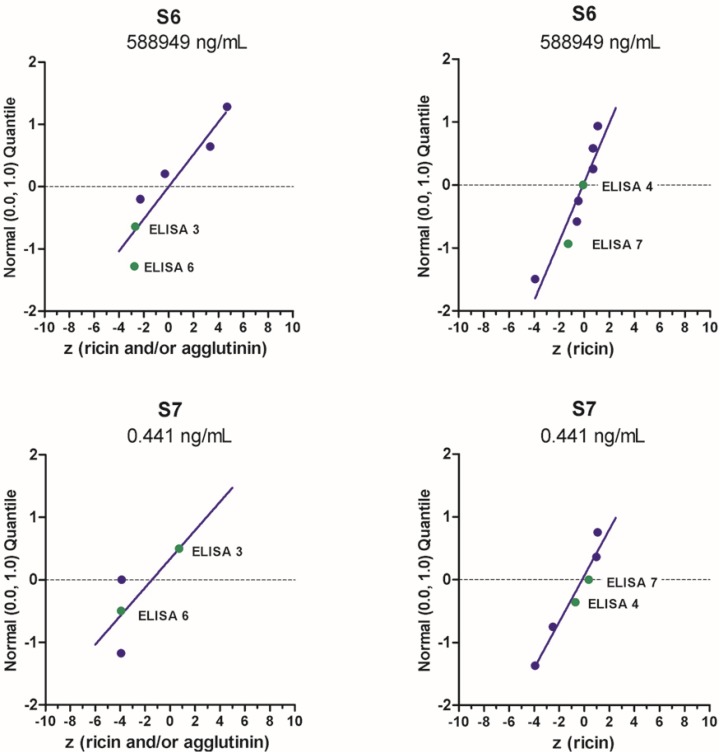
Normal probability plot of *z*-scores for quantification of “ricin” or “ricin and/or RCA120” in samples S6 and S7. Standard normal quantiles were plotted against the *z*-scores (for more information please see [32]). The analysis was done by considering all methods used to quantify the indicated samples. Each dot corresponds to one method used by one laboratory, highlighted in green are results presented by the four different ELISA approaches presented in this manuscript.

Exemplarily, the quantitative results provided by the different ELISA for sample S6 containing the highest concentration of ricin in buffer and sample S7 containing the lowest concentration of ricin is visualized in Figure 4 as normal probability plot of *z*-scores. Overall, ELISA 4 (RKI) and ELISA 7 (Spiez Laboratory) delivered very good results on all or most of the samples tested, as indicated in Figure 4 for samples S6 and S7; all or almost all reported values lay within the interval −2 < *z* < +2 corresponding to satisfactory results. Both laboratories (RKI and Spiez) worked closely together for several years to improve their quality control measures before the actual PT, e.g., by comparing their quantitative data obtained with different in-house reference materials prior to the EQuATox PT. This might have helped in the process of improving their quantification of ricin. ELISA 3 (PHAC-NML) and ELISA 6 (CEA), resulted in *z* scores between 0.6 and −3.9, and the deviation from the assigned value is most probably due to the use of different ricin standard materials (Figure 4).

ELISA 3 developed by PHAC-NML (Canada) quantifies ricin by the interpolation of sample well optical density against a ricin toxin standard curve fit with a 4-parameter logistic (4-PL) model (see Methods). Sample S1, a negative control (0.1% BSA in PBS), was correctly found to contain no ricin or RCA120, and therefore no quantification was possible nor necessary. PHAC quantitative results Samples S4 (ricin in skim milk), S7 (ricin in 0.1% BSA/PBS), S8 (ricin in meat extract), and S9 (organic fertilizer with *R. communis*) had *z*-scores (0.60, 0.74, 0.97, 1.00, respectively) between −2 and 2, indicating a satisfactory result for the quantitation of ricin and RCA120 in each sample. Samples S2 and S5 (RCA120 in 0.1% BSA/PBS) and S3 (ricin in 0.1% BSA/PBS) had the highest *z*-scores (<−3) and were slightly outside of acceptable range for quantitation. Large *z*-scores were obtained for samples containing RCA120-only with no ricin (Samples S2 and S5), and these results are most likely due to the sensitivity and specificity of the ELISA mAbs for ricin toxin.

For ELISA 6 (CEA, France) quantitative results, the sample concentrations containing only RCA120 (S2, S5) were largely underestimated due to the specificity of ELISA 6. For samples S3 (ricin in buffer), S4 (ricin in milk), S6 (high ricin toxin level in buffer), S8 (ricin in meat), a discrepancy around a factor of two was observed between the results and the assigned values. These results are most likely due to the difference of the standards used to assay these samples (reference standard and CEA standard). This difference and the ELISA specificity could explain the large *z*-scores obtained with this ELISA (four samples with a *z*-score < 3 (S2, S5, S7 and S9) and four with −3 < *z*-score < −1 (S3, S4, S6, S8)).

Quantitative results obtained by RKI (Germany) using two different ELISA—one preferentially detecting ricin (ELISA 4a), the other RCA120 (ELISA 4b)—were excellent for both analytes, as *z*-scores all were between the acceptable range of −2 to 2, more precisely between *z* = −0.01 and 1.78. *z*-scores for the high-concentration samples S6 (ricin) and S2 (RCA120) were −0.10 and −0.01, respectively, which is close to an unbiased result of a *z*-score of zero.

Similarly, the quantitative results for ricin obtained by ELISA 7 (Spiez Laboratory, Switzerland) were very good, since all *z*-scores ranged from −1.3 to 0.7. The quantification of ricin in milk and meat was similar to that with buffer, indicating low matrix interference. The quantification of RCA120-containing samples was also good (*z*-scores −1.14 and −1.64) except for sample S9, where a large deviation was observed comparing with the assigned value (*z* = 29.4). This could be explained by the significant cross-reactivity of the RCA120 ELISA in the presence of high amounts of ricin. On the other hand, it was remarkable that quantification of RCA120 delivered convincing results, although a commercially available reference material was used that has been shown to be only 70% pure [21].

## 3. Experimental Section

### 3.1. ELISA 3—Public Health Agency of Canada

Hybridomas producing monoclonal antibodies (mAb) RAC18 (anti-ricin A-chain) and RBC11 (anti-ricin B-chain) were a gracious gift from Dr. Seth Pincus, Louisiana State University [33] and mAbs were produced by the Bioforensics Assay Development and Diagnostic group at the National Microbiology Laboratory as previously described [38]. RAC18 mAb was conjugated to biotin by Rockland Immunologicals (Gilbertsville, PA, USA). Nunclon sterile 96-well flat bottom plates (Sigma-Aldrich, Markham, ON, Canada) were coated with 100 µL per well of 10 µg/mL of RBC11 mAb (in PBS), covered with a plate sealer, and refrigerated overnight at 4 °C. Coated plates were washed four-fold in ELISA wash buffer (0.1% Tween 20 in PBS) using a Delfia Platewash Microtitration Plate Washer (Perkin-Elmer, Woodbridge, ON, Canada). Wells were blocked with 200 µL assay buffer, sealed, and incubated at 37 °C for 60 min. Plates were washed four-fold in ELISA wash buffer. The ricin protein toxin reference material (20 ng/mL), obtained from the Robert Koch Institute (Berlin, Germany) in 2009 and independently prepared from the reference material in this proficiency panel [32], was serially-diluted in duplicate from a single-aliquot working stock in assay buffer and 100 µL was added to each well. BSA (Fisher Scientific, Ottawa, ON, Canada) was used as a negative control in a full dilution series, with equivalent concentrations to the ricin reference material. Sample plate layouts, replicates, and concentrations depended on the type of validation experiment being conducted, but all validation and proficiency panel samples were added at 100 µL per well. Plates were incubated for 60 min at 37 °C, then washed once manually with a multi-channel pipette and filtered tips, then seven-fold with the Delfia plate washer (Perkin-Elmer, Waltham, MA, USA). Biotinylated-RAC18 anti-ricin mAb was diluted to 1 µg/mL in assay buffer, added at 100 µL per well, and incubated at 37 °C for 60 min. Plates were washed seven-fold on the plate washer. Streptavidin-HRP solution (Amersham Biosciences, GE Healthcare, Baie d’Urfe, QC, Canada; 100 µL, diluted 10:1.5 in assay buffer) was added to each well and incubated 37 °C for 45 min. Plates were washed seven-fold on the plate washer, had a final manual wash with PBS and were tapped dry. TMB solution (100 µL, Sigma-Aldrich, Oakville, ON, Canada) was added to each well and incubated at room temperature for 5–10 min. Plates were read on a Victor Wallac3 (Perkin-Elmer, Woodbridge, ON, Canada) at an optical density (OD) of 370 nm. ELISA data was exported to GraphPad Prism 4 software (GraphPad Sofware, La Jolla, CA, USA). ELISA data was normalized by subtracting the overall mean background (BSA) from each sample reading. A 4-PL model was used to fit log-transformed ELISA data, using the following option in GraphPad Prism 4: XY analysis/Nonlinear regression (Curve fit)/Sigmoidal dose-response (Variable slope). Unknown samples had their concentrations interpolated from their OD values by Prism software. Statistical calculations for the mean, standard error (%E), and coefficient of variation (CV%) were processed in Microsoft Excel and regression analysis was conducted in Prism software.

The assay validation study design is described in Tracz *et al.* [34] and is based on previous ELISA validation studies using a 4-PL model [39,40]. Assay performance characteristics were thoroughly evaluated, including measures of accuracy, inter- and intra- assay precision, dilutional linearity, limits of detection and quantitation, and diagnostic sensitivity and specificity. Accuracy, the ability of the assay to be exact in measuring the amount of ricin toxin, was determined by replicate testing of 3 internal quality controls (IQCs, prepared at 1.496, 3.802 and 9.12 ng/mL ricin toxin from RKI, Berlin, Germany), 5 times per plate, in triplicate plates, by two independent operators over 2 non-consecutive days (*n* = 35 each IQC used in final dataset). Intra-assay/intra-operator precision evaluated the variance of IQCs applied to different positions in the ELISA plate. Two independent operators tested 3 IQCs in 4 well locations, in triplicate, on non-consecutive days. Inter-assay/inter-operator precision tests evaluated the repeatability of the ELISA, where two independent operators tested the 3 IQC samples in a minimum of 3 ELISA plates, on non-consecutive days. Inter-assay/intra-operator precision tests evaluated repeatability of the ELISA within a single operator, who tested the IQC samples 5-fold, in a minimum of 3 ELISA plates on non-consecutive days. The limits of quantification were evaluated by testing a series of 9 low-level concentration ricin toxin samples, ranging from below the LOD to above the calculated theoretical LLOQ. These LLOQ validation samples were run by two independent operators, performing the ELISA on non-consecutive days, 7-fold on each plate (*n* = 28 each sample). Dilutional linearity experiments determined if observed assay results were proportional to known ricin toxin concentrations. A range of ricin toxin samples from below the theoretical LLOQ to above the upper asymptote of the reference standard curve were tested by two independent operators, on non-consecutive days, for a total dataset of *n* = 28 each.

### 3.2. ELISA 6—CEA Saclay, France

The production and the selection of monoclonal antibodies anti-ricin A chain and B-chain were previously described [35]. A combinatorial analysis of mAbs was performed to evaluate their simultaneous binding to whole ricin. A two site immunometric test was carried out using one antibody immobilized on solid phase for the capture and another as a biotin-labeled conjugate. The ricin standard used for these experiments was a gracious gift from Dr. Beaumelle (CNRS laboratory, France). The best pair, RB37 mAb (anti-ricin B chain) as capture antibody and RA36 mAb (anti-ricin A chain) as labeled antibody, was used to develop an ELISA detecting both chains of the toxin. RA36 mAb was conjugated with acetycholinesterase (AChE) an enzyme used as tracer in our laboratory [41]. ELISA was performed using Titertek microtitration equipment from Labsystem (Helsinki, Finland), including an automatic washer (Washer 120), an automatic plate reader (Multiskan BICHROMATIC; Thermo Scientific, France). Maxisorp 96-well plates (Nunc, Roskilde, Denmark) were coated with 100 µL per well of 10 µg/mL of RB37 mAb in 50 mM phosphate buffer pH 7.4, covered with a plate sealer. After one night at room temperature, wells were blocked with 300 µL of assay buffer (100 mM phosphate buffer pH 7.4 containing 150 mM NaCl, 0.1% bovin serum albumin and 0.01% sodium azide), sealed and refrigerated at least overnight at 4 °C, until use. The ricin protein toxin standard was purified from castor beans using successive steps: delipidation, affinity purification (galactosyl-Sepharose column, Sigma-Aldrich, France) and cation-exchange chromatography [42]. The ricin protein standard was quantified using its absorbance at 280 nm. We used the ricin isoform D as standard for the ELISA. All dilutions were made in assay buffer (standard, samples, labeled antibody). The ricin protein toxin standard (first concentration at 2 ng/mL in assay buffer) was two-fold serially diluted (8 dilutions in total). 100 µL of standard or diluted samples were added to each well. All standards and samples were assayed using two wells and the assay buffer in eight wells in order to determine the minimum detectable concentration. Each sample was assayed in duplicate. 100 µL of labeled RA36 mAb at 5 UE/mL were added to each well. One UE is defined as the amount of enzyme producing an absorbance increase of one unit per minute and per mL of medium. Plates were incubated at room temperature for 90 min. The plates were washed manually using a multi-channel pipette and filtered tips then 10-fold with the plate washer and were tapped dry. 200 µL of Ellman’s medium (7.5 × 10^−4^ M acetylthiocholine iodide with 5,5-dithiobis 2-nitrobenzoic acid in 0.1M phosphate buffer pH 7.4) were added in each well and incubated at room temperature for 45 min. The optical density of each well was measured at 414 nm. Before the data analysis the optical blank value (mean of the optical density of eight wells containing only Ellman’s medium) was subtracted from each well value. A linear regression was used to fit ELISA data. Unknown sample concentrations were interpolated from their OD values using GraphPad Prism 4 (GraphPad Software, La Jolla, CA, USA). The limit of detection was calculated as the concentration corresponding to the mean of 8 negative controls signal + 3SD. The intra-assay coefficients of variation were determined by assaying five times on the same day five different ricin concentrations (30, 50, 100, 300 and 1500 pg/mL). Inter-assay coefficients were determined by repeating this experiment on six different days.

### 3.3. LFA 4—CEA Saclay, France

We selected the best anti-ricin mAb pairs of the previous combinatorial analysis (see Section 3.2 ELISA) to evaluate their performances in the LFA format. The best sensitivity was obtained with RB37 as capture antibody and RA35 as labeled antibody. The preparation of the test strips and the production of colloidal gold-labeled mAb were previously described [43]. The tests were performed at room temperature in a 96 well-plate by mixing 100 µL/well of samples with 10 µL of a tracer diluted in 0.1 M phosphate buffer pH 7.4 containing 0.1% BSA, 150 mM NaCl and 0.5% Tween. 100 µL of 8 dilutions of the ricin protein standard (200; 100; 50; 20; 10; 5; 2 and 1 ng/mL) were also tested to verify the sensitivity of the LFA. The strips were inserted into the wells to allow the capillary migration of the mixture. After 15 min the signal intensity of the test lines and control lines was qualitatively eye-estimated.

### 3.4. ELISA 4—Robert Koch Institute, Germany

The ricin-specific ELISA (ELISA 4a) was performed as described before in Pauly *et al.*, (2009) using mAb antibody R109 directed against the B-chain of ricin as capture antibody and biotinylated R18 specific for the A-chain as detection tool. Briefly, MaxiSorp microtiter plates were coated with primary mAb (10 µg/mL) in 50 µL PBS overnight at 4 °C and blocked with casein buffer (Senova, Jena, Germany) for 1 h at room temperature. Following washing, 50 µL of toxin was added in serial dilutions from 100 ng/mL to 0.05 pg/mL in assay buffer (PBS, 0.1% BSA (Sigma-Aldrich, Munich, Germany)) and incubated for 2 h at room temperature. The sandwich ELISA was developed by incubation with biotin-labeled secondary antibody diluted in casein buffer (1 h, room temperature), followed by washing and detection with Streptavidin-PolyHRP40 (0.5 ng/mL, Senova, Jena, Germany) and substrate 3,3’,5,5’-tetramethylbenzidine (TMB). Reaction was stopped after 15 min by addition of 100 µL/well 0.25 M H_2_SO_4_ and absorption was determined photospectrometrically at 450 nm (referenced to 620 nm) using a microtiter plate reader (LP400; Anthos Labtec, Wals, Austria).

The RCA120-specific ELISA (ELISA 4b) was performed similarly using mAb ARK4 (kindly provided by Marc-André Avondet, Spiez Laboratory, Switzerland; [36]) as capture antibody and biotinylated polyclonal chicken IgY RC22 [27] as detection antibody.

The half maximal effective concentration (EC_50_) of the ELISA was determined by fitting a four-parametric sigmoidal dose-response equation through the optical density readings over log_10_-transformed antibody concentrations using Prism 5.04 (GraphPad, La Jolla, CA, USA). The intra-assay coefficient of variation was determined by assaying ten times on the same day the EC_50_ concentration. Inter-assay coefficients of variation were determined by measuring 11 ricin concentrations (from 100 ng/mL to 1 pg/mL) on ten consecutive days. In order to determine the working range of the sandwich ELISA, the range in which obtained results have a precision of <20% and a trueness of 80%–120%—a precision plot was generated where CV% inter was plotted against the log ricin concentration. From the graphical display, the upper and lower limit of quantification was derived.

### 3.5. ELISA 7—Spiez Laboratory, Switzerland

The ricin-specific ELISA (ELISA 7a) was performed using mAb RCH1 as capture antibody and the biotinylated mAb1RK1 as detection antibody. Both mAb are directed against the A-chain of ricin. The in-house ricin reference material was manufactured by Uwe Pfüller (University Witten-Herdecke, Institute for Phytochemistry, Witten, Germany) using affinity and size exclusion chromatography. Nunc MaxiSorp microtiter plates (Thermo Scientific, Frankfurt, Germany) were coated with 100 µL per well RCH1 mAb (2.5 mg/mL in carbonate-bicarbonate buffer, pH 9.6). After overnight incubation at 4 °C the plates were washed and blocked with 200 µL blocking buffer (PBS, 1% BSA). After washing, 100 µL (per well) toxin dilutions and sample dilutions were incubated for 2 h at 36 °C. Dilutions were made in assay buffer (PBS, 0.03% BSA, 0.1% Triton-X). The ricin standard was serially two-fold diluted from 500 down to 0.24 ng/mL. After washing, 100 µL of the biotinylated 1RK1 mAb were incubated for 2 h at 36 °C. Following washing 100 µL Streptavidin-HRP Polymer were added and incubated for 1 h at 36 °C. After washing again 100 µL OPD Solution (0.4 mg/mL *O*-Phenylenediamine in 0.05 M Phosphate-Citrate buffer with 0.03% Sodium Perborate, pH 5.0 were incubated for 8.5 min and stopped with 50 µL of 2 M sulfur acid. The absorption was measured at 492 nm using a plate-reader (SPECTRAmax 190, Molecular Devices Ltd., Berks, UK). The data of standards and samples were normalized by subtracting the blank value. A 4-Parameter model was used to fit the data. The concentration of the samples was calculated using SOFTmax PRO 6 Software (Molecular Devices Ltd., Berks, UK).

The RCA120-specific ELISA (ELISA 7b) was performed similarly using an RCA120 specific mAb (ARK4) [36] for capturing combined with the biotinylated 1RK1 mAb for detection. The RCA120 reference material was obtained from Sigma-Aldrich. The RCA120 standard was serially two-fold diluted from 2000 down to 0.98 ng/mL. Data analysis was performed as showed before.

**Table 2:** Quantitative *z*-score analysis of proficiency test panel samples by individual laboratory method. *n*: number of reported participant’s results; *x*: mean of reported participant’s results; *x_a_*: assigned value (unit for S1–S8: ng/mL; for S9: µg/g); *z*: *z*-score.

**Measurand "ricin"**					
**ID Number**	**Sample**	***n***	***x* (ricin)**	*x*_a_ (ricin) *	***z* (ricin)**
ELISA 4	S3	2	442	522	−0.61
ELISA 4	S4	2	393	436	−0.39
ELISA 4	S6	2	574,533	588,949	−0.1
ELISA 4	S7	2	0.36	0.441	−0.71
ELISA 4	S8	2	431	508	−0.59
ELISA 4	S9	2	300	206	1.78
ELISA 7	S1	2	0.015	-	-
ELISA 7	S2	2	993	-	-
ELISA 7	S3	2	438	522	−0.64
ELISA 7	S4	2	294	436	−1.28
ELISA 7	S5	2	1.2	-	-
ELISA 7	S6	2	395,700	588,949	−1.29
ELISA 7	S7	2	0.48	0.441	0.35
ELISA 7	S8	2	438	508	−0.54
ELISA 7	S9	2	239	206	0.61
**Measurand "RCA120"**					
**ID Number**	**Sample**	***n***	***x* (RCA120)**	***x*_a_ (RCA120)** *	***z* (RCA120)**
ELISA 4	S2	2	562,370	563,994	−0.01
ELISA 4	S5	2	436	481	−0.37
ELISA 4	S9	2	46	42	0.32
ELISA 7	S1	2	0	-	-
ELISA 7	S2	2	328,300	563,994	−1.64
ELISA 7	S3	2	96	-	-
ELISA 7	S4	2	0	-	-
ELISA 7	S5	2	341	481	−1.14
ELISA 7	S6	2	83,500	-	-
ELISA 7	S7	2	0	-	-
ELISA 7	S8	2	178	-	-
ELISA 7	S9	2	358	42	29.4
**Measurand "ricin and/or RCA120"**					
**ID Number**	**Sample**	***n***	***x* (ricin A/O RCA120)**	***x*_a_ (ricin A/O RCA120)** *	***z* (ricin A/O RCA120)**
ELISA 3	S1	2	0	-	-
ELISA 3	S2	2	234	572,851	−3.92
ELISA 3	S3	2	79	424	−3.19
ELISA 3	S4	2	725	629	0.6
ELISA 3	S5	2	1.2	445	−3.91
ELISA 3	S6	2	186,451	598,600	−2.7
ELISA 3	S7	2	0.64	0.538	0.74
ELISA 3	S8	2	781	626	0.97
ELISA 3	S9	2	400	318	1
ELISA 6	S1	2	0	-	-
ELISA 6	S2	2	1030	572,851	−3.91
ELISA 6	S3	2	236	424	−1.73
ELISA 6	S4	2	247	629	−2.38
ELISA 6	S5	2	1	445	−3.91
ELISA 6	S6	2	178,849	598,600	−2.75
ELISA 6	S7	2	0	0.538	−3.92
ELISA 6	S8	2	267	626	−2.25
ELISA 6	S9	2	52	318	−3.27

* Please note: Depending on the specificity of assays used by the participants and their reporting (either “ricin”, “RCA120” or “ricin and/or RCA120”), the laboratories’ mean results were slightly different for samples S2–S9. This can be seen when comparing Table 1 with Supporting Table S1 (please see column *x_a_*). Depending on the measurand reported, the assigned values *x_a_* for the nine samples were defined according to the following decision rule [44]: the consensus mean based on the participants’ reported results was used as *x_a_* if the absolute difference between the nominal value determined in the organizer’s laboratory and the mean of the participants’ responses was not larger than 50% of the nominal value given; otherwise the nominal value was used. Therefore, for laboratories which reported their quantitative data as measurand “ricin” or “RCA120”, the values given for *x_a_* and σ***_p_*** from Table 1 were used for calculation of *z*-scores. Otherwise, for laboratories which reported as “ricin and/or RCA120” the values given for *x_a_* and σ***_p_*** from Supporting Table S1 were used for *z*-score calculation.

### 3.6. LFA 2—SPIEZ LABORATORY, Switzerland

Lateral Flow Assay measurements were performed using miPROTECT^®^ Ricin test cartridges, a commercial product by miprolab (Göttingen, Germany). As a positive control ricin standard was diluted in assay-buffer to a concentration of 50 and 100 ng/mL. Samples with higher amounts of ricin or RCA120 were diluted to a concentration of about 100 ng/mL with assay buffer (concentration were estimated from screening tests by ELISA). A negative control was performed with assay buffer. Before charging the test cartridges the samples and standards were diluted 1:1 in test buffer (miprolab). After 20 min the density of the test lines and control lines were evaluated visually.

## 4. Conclusions

This report describes the immunological detection strategies employed by four international laboratories during the 2013 EQuATox ricin proficiency test, focusing on ELISA and LFA tests for the detection of ricin toxin and the highly homologous RCA120. The different ELISA presented in this work turned out to be well-suited to qualitatively detect ricin and/or RCA120 in the sample panel provided. As shown in Figure 3, all ELISAs used by the four laboratories in this proficiency panel correctly detected high- and medium-concentration ricin- and RCA120-containing samples. Additionally, three out of four ELISAs were able to detect sample S7 containing the lowest ricin concentration offered in the PT. Indeed, ELISA-based methods turned out to be the most sensitive detection methods in the international ricin PT [44]. The ELISAs described in this study were able to detect ricin toxin to a lower limit of detection of 2 pg/mL to 0.5 ng/mL. To place this detection value into context, using an inhalational ricin LD_50_ of 5 µg/kg [17] and a 70 kg human being, this amount of ricin toxin is approximately 10^5^ to 10^7^-fold lower than the estimated human lethal dose. Therefore the ELISA tests described in this report can detect ricin at amounts far lower than any “dangerous” sample in the case of a potential bioterrorism threat or suspicious sample.

With respect to ELISA validation, each participant laboratory employed a different approach, with varying sample sizes and experimental design to define assay performance characteristics (see Methods section). Most laboratories used a “sandwich”-type ELISA with monoclonal antibodies specific to the A and B chains of ricin toxin. For data analysis, a 4-parameter logistic model was often used to analyze the data in a non-linear regression curve fit, with varying accuracy and precision tests interpolating sample concentrations from reference curve graphs. In the process of standardization and harmonization of technical approaches, it would be helpful if a common understanding of assay validation would be developed, so that assay parameters could be better compared. As a basic step, the current manuscript is one of a series of method papers in this special issue of Toxins that provides contact points for laboratories seeking experimental advice.

Two laboratories used different LFAs as pre-screening, which correctly detected five or eight sample, respectively. LFAs have an advantage as a front-line, point-of-care type test for rapid detection of ricin toxin. Herein, the LFAs tested could detect ricin in proficiency panel samples containing 206 to 588,949 ng/mL ricin, but not a low-concentration sample with 0.441 ng/mL ricin. Although LFAs are less sensitive for ricin detection than ELISAs, in the context of a rapid point-of-care test they can provide information for the presence of ricin toxin [45]. Due to their lower level of sensitivity, we would recommend any negative results with a high index of suspicion be sent to a reference laboratory for further testing and analysis with a more sensitive methodology. Neither of these immunological approaches (ELISA or LFA) are able to determine the functional activity of ricin which requires separate laboratory tests [5,18,46]. It is important to note that even for LFAs which are advertised as “easy to use assays” a basic level of training and evaluation is necessary before reliable results can be obtained. It has been observed before that depending on the specific LFA used, a significant variability in assay results can be detected: in a previous study, only three out of six commercial LFAs tested on a given sample set performed well [47].

Two of the participant laboratories used two separate ELISAs for the preferential detection of ricin or RCA120, however a clear differentiation into the one or other analyte was still not possible. Both laboratories used mass spectrometry-based approaches to unambiguously determine the analytes in the samples. Therefore, a current limitation of pure immunological approaches is that they cannot unambiguously differentiate the two related analytes. This would require antibodies directed against structurally unique epitopes on the one or other analyte. Alternatively, such clear differentiation of ricin and RCA 120 requires separate laboratory methodology, such as mass spectrometry. In case of an intentional release of toxins from *R. communis*, it is important to detect the material as fast and reliably as possible in order to take adequate actions, and an actual discrimination of ricin and RCA120 is not necessary in the first place. Also depending on the scenario and the material released, such as in the case of a crude castor bean mash preparation, ricin and RCA120 would be found together. In particular cases, such as in the context of identification of chemical weapons by the OPCW or in a case of prosecution, a differentiation of both analytes might be necessary. Here a forensic investigation would require the application of unambiguous identification methods, most probably based on a combination of immunological and mass spectrometric approaches [46,48].

We have highlighted the importance of an international standard material for ricin toxin, allowing for multiple international laboratories to directly compare independently validated immunological assays for ricin detection and identification (see Worbs *et al.* [32]). An assay that quantifies ricin toxin and/or RCA120 with a high level of precision requires: (1) a highly pure and well characterized reference material; (2) highly-specific and high-affinity antibodies (preferably mAbs); (3) thoroughly-validated methodology and standard operating procedures; (4) well-trained laboratory technical personnel for performing methods and generating reproducible results. Quantitative results obtained in the four different labs have shown that it is possible to achieve satisfactory results even in the absence of a commonly available, highly pure reference material; however, it seems to be somewhat arbitrary how close the quantitative data of an individual laboratory ends up with respect to the assigned concentration. Therefore, to improve quality of quantitation, it will be necessary in the future that an thoroughly characterized ricin and RCA120 reference material becomes available for establishing comparable standards worldwide; optimally, certified reference material should be developed. Efforts undertaken in the EU-project EQuATox represent a first step in this direction [32]. Due to the classification of ricin toxin as chemical weapon, we recommend the development of a common international reference material and more standardized assay validation approaches be driven forward by internationally-funded projects like EQuATox, supported by the technological expertise of different international laboratories and standardization bodies.

## Supplementary Materials

Supplementary materials can be accessed at: http://www.mdpi.com/2072-6651/7/12/4858/s1.
