# Supplementary material for: Recommended Immunological Assays to Screen for Ricin-Containing Samples
Source: Toxins (Basel). 2015 Nov 26;7(12):4967–86. doi: 10.3390/toxins7124858 (PMC4690108; doi:10.3390/toxins7124858)
Supplement: Supplementary file 1 [file toxins-07-04858-s001.pdf]

Supplementary Materials

Table S1. Statistics and definition of x_a-values for participants who reported quantitative results as “ricin and/or RCA120”.

Sample	Matrix	Measurand	c(Theoretical) *	c(Nominal) **	σ(Rob)	x_a ***	σ_p	Unit
S1	0.1% BSA/PBS	-	-	-	-	-	-	-
S2	0.1% BSA/PBS	Ricin and/or RCA120	500000	572851	62686	572851	146135	ng/mL
S3	0.1% BSA/PBS	Ricin and/or RCA120	500	504	110	424	108	ng/mL
S4	skimmed milk	Ricin and/or RCA120	500	473	96.3	629	160	ng/mL
S5	0.1% BSA/PBS	Ricin and/or RCA120	500	445	65.2	445	114	ng/mL
S6	0.1% BSA/PBS	Ricin and/or RCA120	500000	589508	78055	598600	152704	ng/mL
S7	0.1% BSA/PBS	Ricin and/or RCA120	0.5	0.414	0.112	0.538	0.137	ng/mL
S8	meat extract	Ricin and/or RCA120	500	484	111	626	160	ng/mL
S9	Organic fertilizer	Ricin and/or RCA120	-	348	-	318	81.2	µg/g

* The “theoretical concentration” was the known concentration of ricin or RCA120 that was spiked into the different matrices. Sample S9 was a naturally contaminated material, the true “theoretical values” were not known; ** Robust estimates of mean nominal concentrations as determined experimentally by the organizing laboratory by ELISA for ricin or RCA120, respectively; *** Consensus mean concentration based on all participants’ reported results as “ricin and/or RCA120” used as “assigned concentration” x_a are highlighted in green; σ(rob): robust estimate of the standard deviation of the nominal concentrations; σ_p: standard deviation for proficiency assessment.
